# Identification of and Associations among Low, Middle, and High Body Composition Trajectories from Age 5- to 17-Years

**DOI:** 10.3390/children7100192

**Published:** 2020-10-20

**Authors:** Teresa A. Marshall, Alexandra M. Curtis, Joseph E. Cavanaugh, John J. Warren, Steven M. Levy

**Affiliations:** 1 Department of Preventive & Community Dentistry, College of Dentistry, The University of Iowa, Iowa City, IA 52242-1010, USA; john-warren@uiowa.edu (J.J.W.); steven-levy@uiowa.edu (S.M.L.); 2 Department of Biostatistics, College of Public Health, The University of Iowa, Iowa City, IA 52242-1010, USA; alexandra-curtis@uiowa.edu (A.M.C.); joe-cavanaugh@uiowa.edu (J.E.C.); 3 Department of Statistics and Actuarial Science, College of Liberal Arts and Sciences, The University of Iowa, Iowa City, IA 52242-1010, USA; 4 Department of Epidemiology, College of Public Health, The University of Iowa, Iowa City, IA 52242-1010, USA

**Keywords:** body mass index, percent body fat, fat-mass index, cluster analyses

## Abstract

Our objective was to identify sex-specific age 5- to 17-year body composition (body mass index (BMI), % body fat, fat mass index, fat-free mass index) trajectories, compare trajectories assigned using age 5 (AGE5) data to those assigned using all available (ALL) data, and compare BMI assignments to other body composition assignments. Cluster analysis was used to identify low, medium, and high trajectories from body composition measures obtained from dual energy x-ray absorptiometry (DXA) scans at 5, 9, 11, 13, 15, and 17 years in a birth cohort followed longitudinally (*n* = 469). Moderate agreement was observed for comparisons between AGE5 data and ALL data cluster assignments for each body composition measure. Agreement between cluster assignments for BMI and other body composition measures was stronger using ALL data than using AGE5 data. Our results suggest that BMI, % body fat, fat mass index, and fat free mass index trajectories are established during early childhood, and that BMI is a reasonable predictor of body composition appropriate to track obesity in public health and clinical settings.

## 1. Introduction

The prevalence of obesity and severe obesity among adults in the United States (US) has continued to increase during the past decade [1]. While the prevalence of obesity has remained stable among US children and adolescents [1,2], the prevalence of severe obesity has increased [2]. According to the 2015–2016 (National Health and Nutrition Examination Survey (NHANES), the prevalence of obesity and severe obesity among US adults was 39.6% and 7.7%, respectively, and among children and adolescents it was 18.5% and 5.6%, respectively [1,2]. Obesity tracks throughout life and is associated with an increased risk of type 2 diabetes, metabolic syndrome, cardiovascular disease, and some cancers [3].

The World Health Organization defines obesity as “abnormal or excessive fat accumulation” [4]. Excess body fat is responsible for the adverse complications associated with obesity, yet is difficult to measure in patient care and population-based studies. Because of its simplicity, the body mass index (BMI), which is based on weight and height measures, is commonly used to identify nutritional status, providing a surrogate measure of overweight or obesity. Alternative measures of nutritional status include percent body fat (%BF), fat-mass index (FMI), and fat-free mass index (FFMI). While % BF adjusts total body fat for body size, % BF does not adjust for lean tissue or height. The FMI and FFMI adjust fat mass and fat-free mass, respectively, for height-squared similar to the BMI, and have been proposed as better indicators of obesity [5].

Weber et al. developed FMI and lean body mass index (LBMI, inclusive of fat-free and bone masses) reference curves for children and adolescents aged 8–20 years using 1999–2004 NHANES data, and reported significant discordance among BMI z-scores, % BF, and FMI [6]. Freedman et al. reviewed associations among BMI, FMI, and FFMI, and reported that BMIs were strongly associated with FMIs in children with BMIs at or above the 85th percentile for age [7]. More recently, Duran et al. reported age- and ethnic-specific reference curves for % BF, FMI, and LBMI for youth aged 8–20 years using 1999–2004 NHANES data [8]. The investigations by Weber et al., Freedman et al., and Duran et al. used cross-sectional data; knowledge of how % BF, FMI, and FFMI track within individuals throughout childhood and adolescence is lacking [6,7,8]. In addition, associations between BMI and other body composition measures throughout childhood and adolescence have not been thoroughly investigated. Understanding how individual body composition measures track throughout childhood and adolescence as well as the associations among the measures is necessary to identify in a timely manner children at risk for obesity. Timely identification of at risk children and/or adolescents will facilitate targeted interventions to arrest further obesity risk, while understanding the general associations between BMI and body composition over time will guide public health prevention strategies.

We addressed these gaps using data from a birth cohort with repeated anthropometric measures and dual-energy X-ray absorptiometry (DXA) scans between ages 5 and 17 years. Our objectives were (1) to identify sex-specific age 5- to 17-year BMI, % BF, FMI, and FFMI trajectories, (2) to compare participants’ trajectory assignments using age 5 year data alone to trajectory assignment using data available for all years, and (3) to compare participants’ BMI trajectory assignment to % BF, FMI, and FFMI trajectory assignments.

## 2. Materials and Methods

### 2.1. Study Participants

Data used in the current prospective cohort analyses were collected as part of the longitudinal Iowa Fluoride (IFS) and Iowa Bone Development Studies (IBDS), Clinical Trial Registration: NCT03547128, which investigated fluoride exposures, dietary intakes, oral health, and bone measures [9,10,11,12,13]. Mothers (*n* = 1882) were recruited at the time of their children’s births for participation in the IFS, and IFS participants active at age 5 (approximately 800) were invited to participate in the IBDS. DXA scans were completed during clinic visits when children were approximately 5, 9, 11, 13, 15, and 17 years of age (*n* = 629 participants with at least one scan, and the following number of participants with DXA scans at each age: 469, 529, 479, 488, 414, and 375, respectively). Inclusion in the age 5 trajectory assignment (*n* = 469) required an age 5 DXA scan, and inclusion in the “all available” trajectory assignment (*n* = 458) required at least four of six possible DXA scans. IFS questionnaires queried family demographic information at birth (1992–1995). Three tiers of socioeconomic status (SES) were defined using household income and mother’s education at baseline. Low SES was defined by a baseline household income <$30,000 and maternal education below a 4-year college degree. Moderate SES was defined by a household income <$30,000 and a maternal education equivalent to a 4-year college degree or higher, or a household income of $30,000–$49,999 and a maternal education below a graduate or professional degree. High SES was defined by a household income of $30,000–$49,999 and a maternal education equivalent to a graduate or professional degree, or a household income of $50,000 or more.

All components of the IFS and IBDS were approved by the Institutional Review Board at the University of Iowa. Written informed consent was obtained from mothers at the time of their child’s birth and from parents at clinic visits, and written assent was obtained from participants beginning at age 13 years.

### 2.2. Anthropometry Measures

Weight (Continental scale, Bridgeview, IL, USA) and height (Harpenden stadiometer, Holtain, UK) were measured by trained and calibrated research nurses during clinic visits. Body mass index (BMI) was calculated as weight/height^2^ (kg/m^2^) [14].

### 2.3. DXA Scans

All scans were completed by one of three experienced research technicians to minimize operator-related variability in the General Clinical Research Center at the University of Iowa using standard positioning [10,11]. Whole body scans at ages 5 and 9 years were completed using a Hologic QDR 2000 model with software version 7.20B and fan-beam mode. Whole body scans at ages 11, 13, 15, and 17 years were completed using Hologic QDR 4500 with software version 12.3 or 12.4 and fan beam mode (Hologic, Watham, MA, USA). Because the Hologic QDR 4500 underestimates fat mass and over-estimates lean mass, age 11, 13, 15, and 17 scans were re-analyzed using the NHANES assessment algorithms [15].

Percent body fat was calculated as (total fat mass/body mass) * 100. FMI was calculated as total fat mass/height^2^ (kg/m^2^). FFMI was calculated as (total mass − fat mass)/height^2^ (kg/m^2^).

### 2.4. Statistical Analyses

Descriptive statistics were calculated for sex-specific anthropometric measures (height and weight) and body composition measures (BMI, % BF, FMI, and FFMI).

Ward’s method of hierarchical clustering, as implemented in the “hclust” function in R, was used to identify sex-specific trajectories for each body composition measure [16]. In order to perform this clustering, the distance or dissimilarity between each pair of participants was calculated. Gower’s method was used to accommodate missing data by using the partial overlap between participants during calculation of these distances using the “daisy” function from the “cluster” package in R [17,18]. The clustering algorithm then assigned each participant to a unique cluster, and iteratively found the cluster merge which optimized Ward’s clustering criteria of maximizing between-cluster variability and minimizing within-cluster variability. This algorithm proceeded until all participants belonged to a single cluster.

Unless data-based criteria or procedures are used to determine the optimal number of clusters, clustering algorithms require the user to either (i) specify a number of clusters a priori, or (ii) use clinical knowledge, coupled with the interpretability and empirical characteristics of the groupings, to determine the most meaningful number of clusters. In this analysis, we considered two to four clusters and chose to work with three clusters for all clustering results. We found the three clusters to be consistently interpretable as high, middle, and low categories, and using the same number of clusters throughout the analysis facilitated comparison across body composition measures.

Clustering was performed separately for males and females in consideration of known growth pattern differences between males and females. Within each sex and body composition measure, two sets of clustering analyses were performed. The first was based on age 5 data only (AGE5; i.e., BMI at AGE5 for males), and the second was based on data available for all years (ALL). The two clustering protocols allowed us to compare participants’ assignment to low, middle, and high trajectories based on AGE5 data to trajectory assignment based on ALL data for each body composition measure.

Because weight and height are more practical clinical measurements than DXA scans, we compared participants’ assignment to low, middle, and high BMI trajectories versus participants’ assignment to low, middle, and high trajectories for % BF, FMI, and FFMI separately based on AGE5 data and ALL data.

The gamma statistic, which ranges from −1 to +1 and can be conceptualized as a correlation coefficient for ordinal variables, was used to quantify the strength of association between two sets of cluster assignments [19].

The concordance-statistic (c-statistic) is a measure of predictive discrimination that ranges from 0 to 1, with higher values representing better discrimination. This statistic was also used to identify agreement between BMI cluster assignments and other body composition measure cluster assignments. The c-statistic represents the percentage of concordant pairs among all pairs of observations. In the present context, a pair of observations is concordant if the observation with the lower BMI cluster assignment also has the lower cluster assignment for the other body composition measure. Therefore, if we observe more concordant pairs, we can conclude that BMI cluster assignment is a good predictor of cluster assignment for other body composition measures.

Despite their different interpretations, the gamma and c-statistics are both based on the number of concordant pairs. The gamma statistic is the difference between the number of concordant and discordant pairs divided by the sum of the number of concordant or discordant pairs. Therefore, whenever the gamma statistic is high, the c-statistic will also be high.

## 3. Results

IFS participants were approximately half female (51.6%) and mostly non-Hispanic white (94.1%). Considering household income and maternal education at baseline, 25.8%, 36.2%, and 38.1% of participants belonged to low, middle, and high SES categories, respectively.

The sample size, age at DXA scan, and median anthropometric and body composition measures, stratified by participant sex and scan age, are provided in Table 1 for participants included in the ALL data cluster (see Table S1 for participants included in the AGE5 data cluster). At each scan, there were slightly more female than male participants. The number of participants with DXA scans at each age was relatively stable in the ALL data cluster, because we required participants to have four out of six exams for inclusion in this analysis. Weight and BMI generally increased with age, while height initially increased and then gradually leveled off for females. Percent BF increased steadily for females, while % BF initially increased and then decreased after 11 years for males. The trend for FFMI is similar to the trend for BMI and the trend for FMI is similar to the trend for % BF.

**Figure 1:**
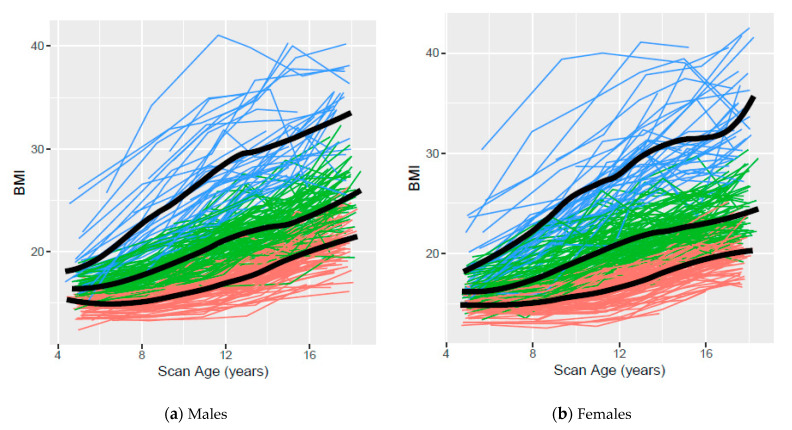
Low, middle, and high body mass index (BMI) clusters using data available for all years (ALL) from ages 5 to 17 years for Iowa Bone Development Subjects (*n* = 458, 223 males and 235 females). Orange, green, and blue lines represent individuals assigned to low, middle, and high clusters, respectively.

Figure 1 presents the observed age 5- to 17-year BMI trajectories based on clustering using ALL data for males and females, respectively, with colors indicating cluster assignment and the smoothed mean for each cluster overlaid on the observed data. The smoothed mean trajectory of the high BMI cluster appears to increase slightly more rapidly than the trajectories of the low or middle BMI clusters for both males and females.

**Figure 2:**
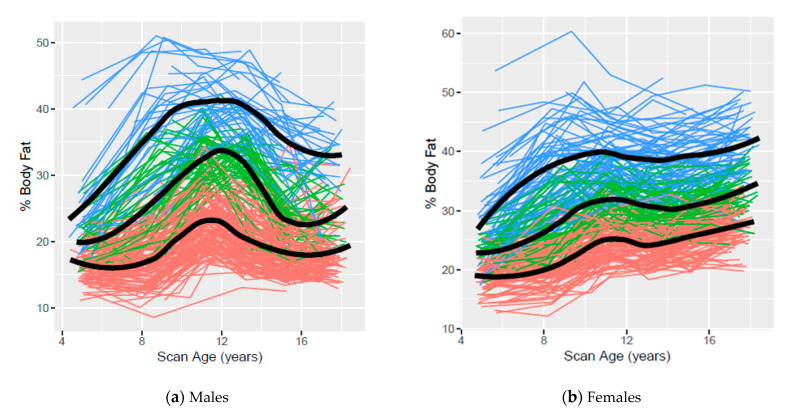
Low, middle and high body percent body fat clusters using ALL data from ages 5 to 17 years for Iowa Fluoride Subjects (*n* = 458, 223 males and 235 females). Orange, green, and blue lines represent individuals assigned to low, middle, and high clusters, respectively.

Figure 2 presents the observed age 5- to 17-year % BF trajectories based on clustering using ALL data for males and females, respectively, with colors indicating cluster assignment and the smoothed mean for each cluster overlaid on the observed data. Percent BF trajectories for males increased throughout childhood, declined in early adolescence and appeared to stabilize in late adolescence, while % BF trajectories generally increased for females throughout childhood and adolescence.

**Figure 3:**
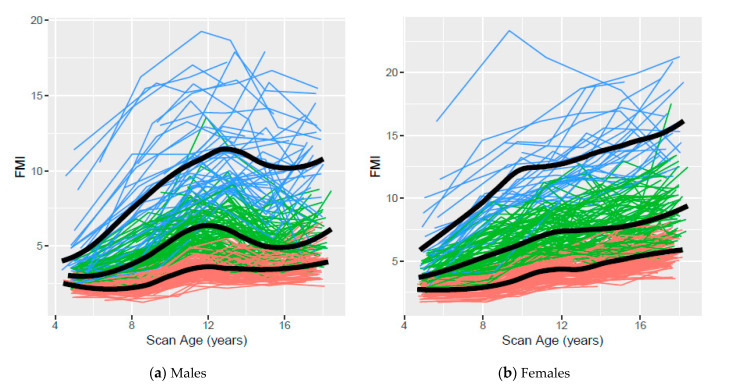
Low, middle, and high fat mass index (FMI) clusters using ALL data from ages 5 to 17 years for Iowa Fluoride Subjects (*n* = 458, 223 males and 235 females). Orange, green, and blue lines represent individuals assigned to low, middle, and high clusters, respectively.

Figure 3 presents the observed age 5- to 17-year FMI trajectories based on clustering using ALL data for males and females, respectively, with colors indicating cluster assignment and the smoothed mean for each cluster overlaid on the observed data. FMI trajectories mirrored % BF trajectories for both sexes. They increased for males throughout childhood, declined in early adolescence and appeared to stabilize in late adolescence, while FMI trajectories for females generally increased throughout childhood and adolescence.

**Figure 4:**
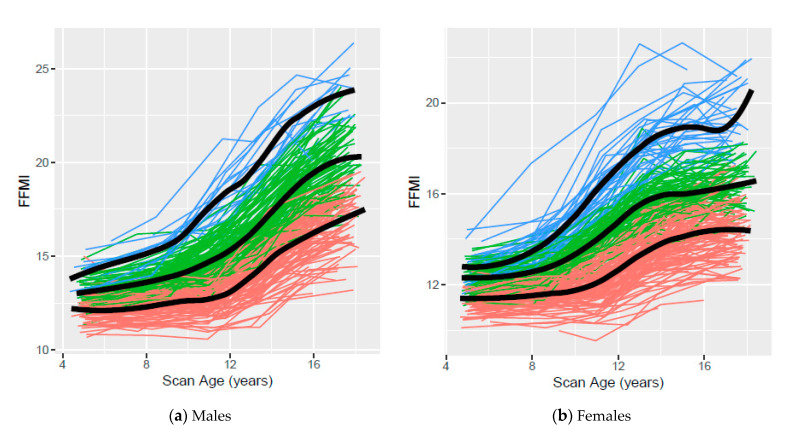
Low, middle, and high fat free mass index (FFMI) clusters using ALL data from ages 5 to 17 years for Iowa Fluoride Subjects (*n* = 458, 223 males and 235 females). Orange, green, and blue lines represent individuals assigned to low, middle, and high clusters, respectively.

Figure 4 presents the observed age 5- to 17-year FFMI trajectories based on clustering using ALL data for males and females, respectively, with colors indicating cluster assignment and the smoothed mean for each cluster overlaid on the observed data. FFMI trajectories increased more rapidly throughout adolescence for males, and more rapidly during late childhood than adolescence for females. Median body composition measures for low, middle, and high clusters based on AGE5 and ALL data, respectively, are available online (see Table S2). Trajectories based on clustering using AGE5 data are available online (see Figures S1–S4).

Comparisons of cluster assignments using AGE5 data and using ALL data for body composition measures are shown in Table 2. Gamma statistics for trend, which quantify the strength and reflect the direction of monotonic associations between two ordinal variables, range from 0.77 for % BF (males) to 0.92 for FMI (females). These results suggest that assignment based on AGE5 data alone provides similar classifications to assignment based on ALL measurements throughout childhood and adolescence for BMI, % BF, FMI, and FFMI.

The comparisons between participant assignment to BMI clusters and assignment to % BF, FMI, and FFMI clusters are shown in Table 3. Results are provided for gamma statistics and c-statistics, and the results of these two measures generally agree. Overall, there was a strong association between BMI cluster assignments and % BF, FMI, and FFMI assignments using ALL data (gamma statistics ranging from 0.90 to 0.96, c-statistics ranging from 0.84 to 0.88). Both measures of association are less for comparisons between clusters assigned using AGE5 data than for clusters assigned using ALL data for BMI—FFMI (females), BMI—% BF (males) and BMI—FMI (males). Gamma statistics and c-statistics are generally less for comparisons between BMI clusters assigned using AGE5 data and % BF, FMI, and FFMI clusters assigned using ALL data, relative to clusters assigned using ALL data.

Summary: Low, medium, and high trajectories were identified for BMI, % BF, FMI, and FFMI based on clustering using AGE5 or ALL data. Trajectories generally increased with age, although differences between genders were noted. Assignment to BMI, % BF, FMI, and FFMI clusters using AGE5 data was similar to cluster assignments using ALL data for both genders. In addition, assignment to BMI clusters was generally similar to % BF, FMI, and FFMI cluster assignments, although the agreement was stronger for ALL data compared to AGE5 data.

## 4. Discussion

We identified how body composition measures track within individuals followed longitudinally throughout childhood and adolescence and explored associations between BMI and other body composition measures during this time frame.

Low, middle, and high trajectories for each body composition measure based on clustering were identified using AGE5 or ALL data. The variability and overlap among participant trajectories within and between clusters observed in all scatterplots confirms that body composition measures are dynamic and change with age. Although overlap among participants from different clusters was observed, our trajectories suggest that distinct patterns exist. In general, less overlap was observed in body composition measures based on clusters assigned using ALL data than those assigned using AGE5 data, emphasizing the value of multiple data points.

Agreement between cluster assignments (i.e., low, middle, or high) using AGE5 or ALL data was good for BMI and each body composition measure, suggesting that one’s BMI and relative distribution of % BF, FMI, and FFMI are established during early childhood and remain stable throughout adolescence. These data are consistent with observations that children having high BMIs during childhood are at increased risk of obesity during adolescence and adulthood [20,21,22,23]. Simmonds et al. conducted a systematic review and meta-analyses to investigate the persistence of obesity from childhood into adulthood, and reported a strong positive association between a high childhood BMI and adult obesity [20]. They also noted that use of childhood BMI to predict adult obesity has limitations, as most obese adults are not obese as children. Freedman et al. reported similar findings from the Bogalusa Heart study after following 3096 school age children into adulthood [21]. Of the children whose BMI exceeded 120% of their age- and sex-specific 95th BMI percentile, 69% had a BMI greater than or equal to 40 during adulthood. Yet, 55% of adults whose BMI was greater than or equal to 40 had childhood BMIs below the 95th percentile. Using growth data from German children recorded in the CrescNet patient registry, Geserick et al. found that preschool children with high acceleration in annual BMI increments were 1.4 times as likely to be overweight or obese during adolescence as their peers with stable preschool BMIs [22]. Evensen et al. reported that high BMIs during early childhood predicted higher fat mass during late adolescence [23]. These findings extend previous BMI findings by documenting that % BF, FMI, and LMI also track within individuals throughout childhood and adolescence.

Differences in agreement between BMI cluster and % BF, FMI, and FFMI cluster assignments observed between sexes using AGE5 data reflect the natural variation between FMI and FFMI between sexes. Girls had higher % BF and FMI than boys at AGE5; therefore, % BF and FMI agreed more with BMI for girls than for boys. Similarly, FFMI was higher in boys at AGE5 and better agreement was noted between FFMI and BMI in boys than in girls.

For both sexes, cluster agreement between BMI and body composition measures improved when ALL data were used. The consistency observed between BMI and % BF, FMI, and FFMI cluster assignments using ALL data suggest that BMI tracks with other body composition measures. Agreement between BMI cluster assignment using AGE5 data and other body composition measures using ALL data was generally less than for only AGE5 or only ALL data. However, moderate agreement was observed, suggesting that in an absolute sense, AGE5 BMI is a reasonable predictor of adolescent body composition measures. This finding extends previous knowledge by documenting that an early BMI measure may be useful to predict adolescent body composition.

Additional studies will be required to confirm our findings, and extend investigation of body composition trajectory identification throughout adulthood. Identification of trajectories for body composition measures will facilitate investigation of behavioral factors influencing trajectory membership as well as clinical trials to identify therapeutic measures for obesity treatment.

While our objective was to identify BMI and other body composition trajectories, compare trajectories based on one to four or more time points, and consider associations among measures, the implications of our findings for clinical practice deserve mention. We demonstrated that BMI, % BF and FMI trajectories are established during early childhood, suggesting that dietary, activity, and other behavioral interventions targeting obesity should be employed early in children with obesity. We demonstrated that BMI trajectories are consistent with % BF and FMI trajectories, suggesting that BMI is an appropriate measure in public health and clinical settings to identify children with obesity. Furthermore, BMI measures are appropriate in public health settings to monitor outcomes of obesity preventive programs.

Both strengths and weaknesses should be considered when interpreting the current results. The IFS/IBDS is in a unique position to evaluate longitudinal BMI and body composition measures across childhood and adolescence. Longitudinal studies are widely known to reduce the cohort effect, which occurs when participants who share a similar birth year (or other shared experience) differ from those in a later birth year. While cross-sectional studies may suffer from a cohort effect due to changes in the obesity rates among U.S. children, cohort effects do not have much of an impact on the longitudinal IFS. However, the relatively small sample is self-selected, mostly white, reasonably well-educated, reasonably high socioeconomic status, and not representative of other US or international populations. Additionally, we used two different machines due to changing technology over the long follow-up period—the first for the measurements at ages 5 and 9 years, and the second for age 11 years and after.

There are a wide variety of clustering methods described in the statistics literature, each with different advantages and disadvantages. Although we initially considered latent class mixture models, which take into account the temporal nature of our data and are able to better accommodate missing values, most body composition measures in this analysis (with the possible exception of BMI) are not linear over time, and many have a skewed distribution at most exam ages. These additional complications with regard to model fitting and interpretation led us to utilize clustering methods that do not rely on mixed modeling. Within these clustering methods, utilization of Gower’s distance measures allowed us to accommodate some missing data (i.e., participants with at least 4 out of 6 DXA scans for the ALL data clusters), but not as much missing data as the latent class mixture models may have accommodated. Although Ward’s method of clustering has a limitation of being a greedy algorithm that relies on the user to select the number of clusters, we felt we were able to identify a clinically meaningful number of clusters and the goal of minimizing the within-cluster variance is a reasonable objective given our data set.

## 5. Conclusions

We described and compared body composition measure trajectories based on clustering using only AGE5 data and ALL data available between ages 5 and 17 years. Body mass index, % BF, FMI, and FFMI tracked throughout childhood and adolescence for both males and females. While measurements at multiple time points are desirable, a single, early time point may be considered predictive of body composition trajectory in the absence of multiple data points or before multiple data points can be gathered. Our results support early intervention in children identified as obese or at risk for obesity. Body mass index is a reasonable predictor of body composition, and appropriate in both public health and clinical settings to track obesity.

## Figures and Tables

**Table 1:** Median (25th, 75th percentile) body composition measures of Iowa Bone Development Study participants (*n* = 458) at each scan age.

Body Composition Measure	Scan Age											
	5 Years		9 Years		11 Years		13 Years		15 Years		17 Years	
	Male	Female	Male	Female	Male	Female	Male	Female	Male	Female	Male	Female
Number	172	199	214	229	208	221	211	222	193	201	158	190
Actual Age (years)	5.1 (4.9, 5.5)	5.2 (4.9, 5.5)	8.6 (8.2, 9.2)	8.6 (8.3, 9.1)	11.1 (11.0, 11.4)	11.2 (11.0, 11.4)	13.2 (13.0, 13.5)	13.2 (13.0, 13.5)	15.3 (15.1, 15.6)	15.2 (15.1, 15.5)	17.6 (17.2, 17.9)	17.5 (17.2, 17.8)
Weight (kg)	20.0 (18.3, 21.5)	19.3 (17.6, 21.4)	31.3 (26.8, 37.6)	30.0 (25.9, 36.1)	42.0 (36.0, 51.0)	42.1 (35.2, 51.4)	55.8 (45.9, 66.5)	53.0 (45.6, 63.3)	67.4 (59.6, 76.5)	58.0 (51.6, 67.9)	75.9 (65.9, 87.2)	63.0 (55.1, 74.3)
Height (cm)	111.9 (108.6, 115.3)	110.4 (107.6, 115.3)	134.3 (130.0, 139.4)	133.0 (128.1, 137.6)	148.5 (143.8, 154.5)	149.4 (143.9, 154.0)	163.0 (155.9, 169.5)	161.2 (156.9, 164.6)	174.9 (170.6, 181.2)	164.6 (159.4, 169.0)	179.1 (173.6, 184.0)	165.7 (161.5, 170.3)
Body Mass Index	15.9 (15.1, 16.8)	15.7 (14.8, 16.8)	17.1 (15.7, 19.5)	16.9 (15.4, 19.4)	19.1 (16.8, 22.0)	18.8 (16.6, 22.1)	20.7 (18.1, 24.0)	20.5 (18.1, 24.1)	21.7 (19.9, 24.9)	21.5 (19.5, 24.7)	23.8 (21.0, 27.3)	22.5 (20.6, 26.7)
% Body Fat	17.6 (15.6, 20.3)	21.4 (18.9, 25.8)	20.9 (16.4, 28.4)	25.9 (20.7, 33.9)	27.3 (22.1, 33.6)	29.6 (25.7, 35.6)	23.6 (18.8, 33.5)	28.8 (24.5, 34.6)	20.2 (16.8, 24.8)	29.9 (26.1, 35.5)	19.7 (16.6, 26.4)	31.7 (27.8, 37.8)
Fat Mass Index	2.8 (2.3, 3.3)	3.3 (2.7, 4.0)	3.4 (2.5, 5.2	4.2 (3.1, 6.4)	5.2 (3.7, 7.3)	5.5 (4.4, 7.9)	4.6 (3.5, 8.0)	6.0 (4.4, 8.3)	4.3 (3.4, 6.2)	6.6 (5.1, 8.7)	4.5 (3.6, 6.9)	7.1 (5.7, 10.0)
Fat-Free Mass Index	12.6 (12.1, 13.2)	11.9 (11.4, 12.4)	13.2 (12.5, 14.0)	12.2 (11.6, 13.0)	13.8 (12.9, 15.1)	13.4 (12.2, 14.6)	15.4 (14.2, 16.9)	14.7 (13.4, 16.1)	17.3 (15.9, 19.0)	15.3 (14.2, 16.5)	18.5 (17.2, 20.5)	15.7 (14.4, 17.2)

**Table 2:** Comparisons between cluster assignments based on AGE5 data and ALL data for body composition measures of Iowa Bone Development Study participants (*n* = 458).

Body Composition Measure	Females	Males
	Gamma Statistic for Trend *	Gamma Statistic for Trend
Body Mass Index	0.82 (0.74, 0.90)	0.84 (0.75, 0.93)
% Body Fat	0.86 (0.78, 0.93)	0.77 (0.64, 0.90)
Fat Mass Index	0.92 (0.87, 0.97)	0.82 (0.71, 0.93)
Fat-Free Mass Index	0.86 (0.78, 0.93)	0.88 (0.81, 0.96)

* Gamma statistic for trend (95% confidence interval calculated using the standard estimated asymptotic standard error); AGE5: age 5

**Table 3:** Comparisons between cluster assignments for body mass index trajectory and % body fat, fat mass index and fat-free mass index trajectory based on AGE5 year data and ALL data for participants of the Iowa Bone Development Study (*n* = 458).

Body Composition Measure	Females						Males					
	AGE5 BMI Cluster and AGE5 Body Composition Cluster Assignment		ALL Data BMI Cluster and ALL Data Body Composition Cluster Assignment		AGE5 BMI Cluster and ALL Data Body Composition Cluster Assignment		AGE5 BMI Cluster and AGE5 Body Composition Cluster Assignment		ALL Data BMI Cluster and ALL Data Body Composition Cluster Assignment		AGE5 BMI Cluster and ALL Data Body Composition Cluster Assignment	
	Gamma Statistic for Trend *	c-Statistic	Gamma Statistic for Trend	c-Statistic	Gamma Statistic for Trend	c-Statistic	Gamma Statistic for Trend	c-Statistic	Gamma Statistic for Trend	c-Statistic	Gamma Statistic for Trend	c-Statistic
% Body Fat	0.91 (0.85, 0.97)	0.84	0.92 (0.87, 0.96)	0.84	0.77 (0.67, 0.87)	0.77	0.78 (0.65, 0.90))	0.74	0.90 (0.84, 0.97)	0.84	0.66 (0.51, 0.81)	0.70
Fat Mass Index	0.97 (0.94, 1.00)	0.88	0.96 (0.93, 0.99)	0.88	0.86 (0.78, 0.94)	0.81	0.90 (0.83, 0.98)	0.79	0.95 (0.91, 0.98)	0.88	0.70 (0.58, 0.82)	0.71
Fat-Free Mass Index	0.82 (0.74, 0.90)	0.79	0.96 (0.93, 0.99)	0.88	0.84 (0.75, 0.92)	0.80	0.96 (0.93, 0.99)	0.82	0.91 (0.86, 0.97)	0.85	0.94 (0.88, 1.00)	0.81

* Gamma statistic for trend (95% confidence interval calculated using the standard estimated asymptotic standard error). BMI: body mass index

## Supplementary Materials

The following are available online at https://www.mdpi.com/2227-9067/7/10/192/s1, Figure S1: Low, middle, and high body mass index clusters using AGE5 year data for Iowa Fluoride Subjects., Figure S2: Low, middle and high body percent body fat clusters using AGE5 year data for Iowa Fluoride Subjects., Figure S3: Low, middle, and high fat mass index clusters using AGE5 year data for Iowa Fluoride Subjects., Figure S4: Low, middle, and high fat free mass index clusters using AGE5 year data for Iowa Fluoride Subjects., Table S1: Median (25th, 75th percentile) body composition measures of Iowa Bone Development Study participants in the AGE5 cluster analysis at each scan age. Table S2: Sample sizes and medians (25th, 75th percentile) for body mass index, % body fat, fat mass index and fat-free mass index at each scan age for clusters assigned using either only AGE5-year or ALL data.
