# Supplementary material for: Identification of and Associations among Low, Middle, and High Body Composition Trajectories from Age 5- to 17-Years
Source: Children (Basel). 2020 Oct 20;7(10):192. doi: 10.3390/children7100192 (PMC7589976; doi:10.3390/children7100192)

Supplemental Table 1: Median (25th, 75th percentile) body composition measures of Iowa Bone Development Study participants in the age 5 cluster analysis (n= 469) at each scan age.

**Body composition measure**	**Scan Age**											
	5 years		9 years		11 years		13 years		15 years		17 years	
	Male	Female	Male	Female	Male	Female	Male	Female	Male	Female	Male	Female
Number	218	251	193	222	172	208	169	189	145	172	117	158
Actual Age (years)	5.1 (5.0,5.5)	5.2 (5.0,5.6)	8.6 (8.2,9.0)	8.6 (8.2,9.0)	11.1 (11.0,11.4)	11.2 (11.0,11.4)	13.2 (13.0,13.5)	13.2 (13.0,13.5)	15.3 (15.1,15.6)	15.2 (15.1,15.5)	17.5 (17.1,17.8)	17.5 (17.2,17.8)
Weight (kg)	19.9 (18.3,21.5)	19.3 (17.7,21.5)	30.9 (26.3,36.7)	29.9 (25.7,36.0)	42.6 (36.1,50.5)	41.7 (35.1,51.3)	56.2 (45.9,66.4)	53.0 (45.6,63.4)	68.0 (59.5,76.5)	57.5 (51.4,68.2)	77.0 (66.3,87.7)	62.5 (54.2,74.6)
Height (cm)	111.8 (108.2, 115.3)	110.2 (107.4, 114.8)	133.6 (129.6, 138.7)	132.4 (127.7, 136.9)	149.0 (144.1, 154.5)	148.5 (143.1, 153.4)	163.2 (156.4, 170.1)	160.7 (156.3, 164.4)	175.2 (170.2, 181.8)	164.2 (159.0, 168.6)	179.4 (173.3, 184.1)	165.5 (161.5, 170.1)
Body Mass Index	15.9 (15.1,16.9)	15.8 (14.9,16.8)	17.0 (15.6,19.1)	17.0 (15.4,19.4)	18.9 (16.6,21.9)	19.0 (16.6,22.3)	20.9 (18.1,23.7)	20.8 (18.2,24.0)	21.6 (19.9,24.6)	21.5 (19.6,24.7)	23.8 (21.0,27.3)	22.6 (20.6,26.7)
% Body Fat	17.7 (15.7,20.6)	21.4 (19.0,25.6)	20.5 (16.4,25.7)	25.8 (20.7,33.8)	26.9 (21.8,33.3)	29.9 (25.6,36.3)	23.6 (18.6,32.4)	28.9 (24.6,34.5)	20.4 (16.8,25.3)	29.9 (26.1,35.4)	19.5 (16.6,26.5)	31.4 (27.8,37.9)
Fat Mass Index	2.8 (2.3,3.3)	3.3 (2.8,4.0)	3.4 (2.5,4.7)	4.2 (3.1,6.4)	5.2 (3.6,7.2)	5.5 (4.4,8.1)	4.6 (3.5,7.7)	6.0 (4.4,8.2)	4.4 (3.4,6.2)	6.6 (5.1,8.9)	4.5 (3.6,6.9)	7.0 (5.7,10.0)
Fat-Free Mass Index	12.6 (12.1,13.2)	11.9 (11.4,12.4)	13.1 (12.4,13.8)	12.3 (11.7,13.0)	13.8 (12.8,15.1)	13.4 (12.2,14.7)	15.6 (14.2,17.0)	14.8 (13.5,16.1)	17.2 (15.8,19.0)	15.3 (14.2,16.5)	18.6 (17.1,20.6)	15.7 (14.5,17.2)

Supplemental Table 2: Sex-specific sample sizes and medians (25th,75% percentiles) for body mass index, % body fat, fat mass index and fat-free mass index at each scan age for clusters assigned using either only age 5-year or all available data (n=469, 218 males and 251 females for age 5 clustering; n=458, 223 males and 235 females for all available data clustering).

**Males**	**Cluster**	**Scan Age**					
		5 years	9 years	11 years	13 years	15 years	17 years
Body Mass Index							
Age 5 Data: Sample size, Median (25th, 75th percentile)	Low	46	39	38	36	34	28
		14.5 (14.0,14.8)	14.8 (14.1,15.5)	15.7 (14.9,16.8)	16.7 (15.9,18.4)	18.8 (17.8,20.5)	20.0 (18.3,22.5)
	Middle	128	112	100	96	83	67
		15.9 (15.5,16.4)	17.0 (16.0,18.4)	18.9 (17.2,21.4)	20.8 (18.4,23.3)	21.9 (20.3,24.6)	24.3 (21.9,27.6)
	High	44	42	34	37	28	22
		18.2 (17.7,19.2)	20.7 (19.1,24.6)	23.6 (21.4,29.2)	26.0 (22.1,29.2)	24.8 (22.9,28.7)	26.3 (25.1,31.0)
All Available Data Sample size, Median (25th, 75th percentile)	Low	68	84	81	84	80	66
		15.1 (14.5,15.7)	15.3 (14.6,16.0)	16.4 (15.7,17.0)	17.9 (16.6,18.4)	19.4 (18.4,20.9)	21.0 (19.5,22.8)
	Middle	75	92	90	90	81	69
		16.2 (15.7,17.1)	18.2 (17.1,19.3)	20.2 (18.7,21.7)	21.8 (20.5,23.3)	22.9 (21.1,24.5)	24.8 (23.1,27.3)
	High	29	38	37	37	32	23
		17.9 (16.6,19.1)	22.4 (21.1,26.6)	26.7 (24.0,31.6)	27.7 (26.9,33.8)	29.3 (27.5,33.0)	34.0 (30.0,35.6)
% Body Fat							
Age 5 Data Sample size, Median (25th, 75th percentile)	Low	142	126	115	110	98	80
		3.0 (2.6,3.4)	4.8 (3.6,6.8)	9.1 (7.4,12.8)	10.6 (8.0,15.8)	11.9 (9.9,15.7)	13.1 (10.5,19.4)
	Middle	69	61	53	54	45	35
		4.3 (3.9,5.1)	9.5 (6.3,13.2)	17.0 (12.0,20.6)	17.2 (12.5,26.5)	18.3 (13.4,24.9)	21.9 (14.5,29.3)
	High	7	6	4	5	2	2
		13.4 (12.6,16.0)	30.3 (29.3,32.6)	39.8 (37.2,44.9)	45.4 (40.8,49.9)	42.6 (32.5,52.8)	41.4 (31.7,51.2)
All Available Data (Sample size, Mean (SD), Median (25th, 75th percentile))	Low	106	126	126	126	115	97
		3.0 (2.6,3.6)	4.7 (3.6,6.1)	8.5 (7.2,11.1)	9.7 (8.0,11.8)	11.0 (9.4,13.3)	12.0 (10.1,14.9)
	Middle	39	52	49	51	47	36
		4.1 (3.4,4.8)	9.9 (6.8,12.1)	15.9 (13.4,19.5)	19.7 (15.7,23.4)	16.6 (13.7,20.7)	19.9 (14.7,24.4)
	High	27	36	33	34	31	25
		4.6 (3.9,6.3)	14.4 (10.7,22.8)	23.1 (19.1,34.2)	29.8 (26.3,37.1)	32.0 (24.7,38.5)	32.4 (27.3,41.4)

Fat Mass Index							
Age 5 Data Sample size, Median (25th, 75th percentile)	Low	91	79	71	69	60	51
		2.2 (2.1,2.4)	2.5 (2.0,3.0)	3.6 (3.1,4.6)	3.5 (2.9,4.3)	3.5 (2.9,4.6)	3.6 (3.1,4.6)
	Middle	120	108	97	95	83	64
		3.1 (2.8,3.5)	4.0 (3.3,5.8)	6.2 (5.1,8.3)	5.8 (4.2,9.1)	4.9 (3.9,7.7)	5.3 (4.2,8.7)
	High	7	6	4	5	2	2
		10.6 (8.7,11.4)	14.8 (14.1,15.8)	16.6 (15.7,18.1)	15.8 (15.7,17.2)	14.8 (12.9,16.7)	13.8 (12.1,15.5)
All Available Data Sample size, Median (25th, 75th percentile)	Low	70	85	84	86	80	71
		2.2 (2.1,2.6)	2.3 (2.0,2.8)	3.6 (3.1,3.9)	3.4 (3.0,3.9)	3.4 (2.9,3.9)	3.6 (3.1,4.1)
	Middle	67	84	82	83	75	57
		2.9 (2.6,3.4)	4.0 (3.4,5.2)	5.9 (5.2,6.9)	5.7 (4.3,7.2)	4.8 (3.9,5.7)	5.1 (4.3,6.6)
	High	35	45	42	42	38	30
		3.5 (3.1,4.9)	7.7 (5.1,10.2)	9.9 (8.7,12.8)	10.9 (9.2,12.3)	9.8 (7.7,12.1)	10.8 (9.5,12.1)
Fat-Free Mass Index							
Age 5 Data Sample size, Median (25th, 75th percentile)	Low	48	41	38	36	35	23
		11.6 (11.4,11.9)	11.9 (11.4,12.4)	12.2 (11.8,12.9)	13.5 (12.6,14.4)	15.4 (14.3,15.9)	15.9 (14.4,18.3)
	Middle	83	75	66	65	54	45
		12.4 (12.2,12.6)	13.0 (12.5,13.4)	13.5 (12.9,14.3)	15.3 (14.2,16.4)	17.1 (16.1,18.2)	18.6 (17.6,19.7)
	High	87	77	68	68	56	49
		13.5 (13.1,13.9)	14.0 (13.5,14.7)	15.2 (14.2,16.2)	16.8 (15.9,18.5)	19.0 (17.2,20.6)	20.3 (18.3,21.9)
All Available Data Sample size, Median (25th, 75th percentile)	Low	85	102	100	99	97	79
		12.2 (11.8,12.4)	12.4 (12.0,12.9)	12.9 (12.2,13.3)	14.1 (13.3,15.0)	15.9 (15.1,16.8)	17.3 (15.9,18.3)
	Middle	74	94	90	96	83	69
		13.0 (12.7,13.6)	13.8 (13.4,14.2)	14.6 (14.0,15.5)	16.5 (15.6,17.3)	18.7 (17.9,19.7)	20.1 (19.2,21.6)
	High	13	18	18	16	13	10
		14.3 (13.7,14.5)	15.5 (15.1,16.0)	17.9 (17.3,18.5)	19.4 (18.6,20.8)	22.3 (21.6,23.3)	24.0 (22.5,24.7)

Supplemental Figure 1. Low, middle, and high body mass index clusters using age 5 year data for Iowa Bone Development Study Subjects (n=469, 218 males and 251 females). Orange, green, and blue lines represent individuals assigned to low, middle, and high clusters, respectively.

Figure 1a – Males Figure 1b – Females

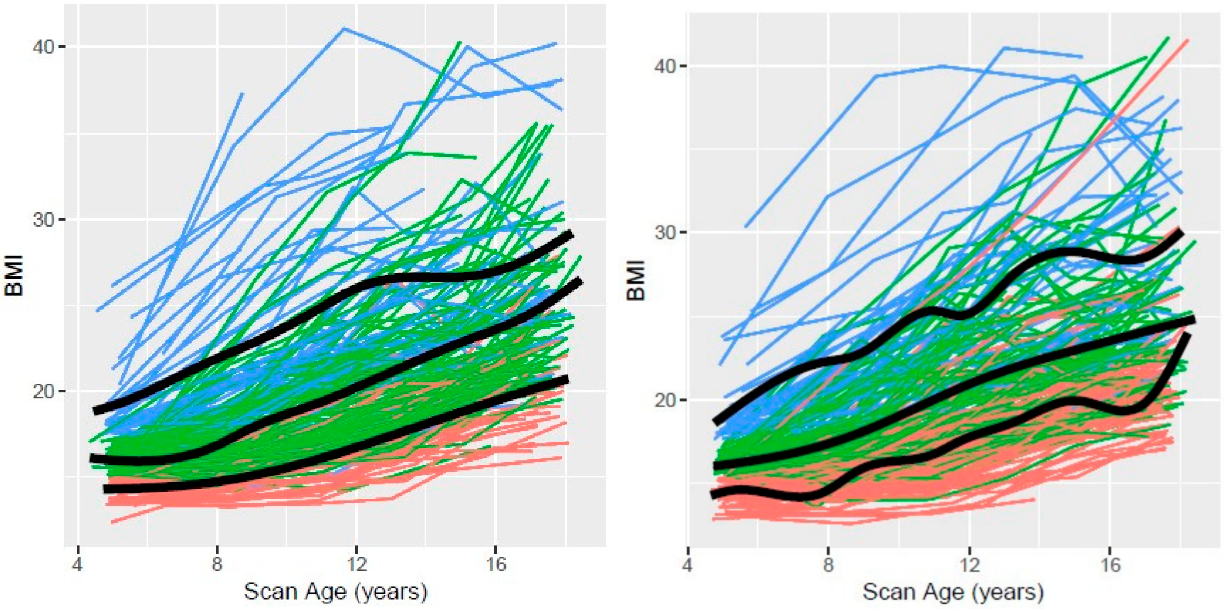

Supplemental Figure 2. Low, middle and high body percent body fat clusters using age 5 year data for Iowa Bone Development Study subjects (n=469, 218 males and 251 females). Orange, green, and blue lines represent individuals assigned to low, middle, and high clusters, respectively.

Figure 2a – Males

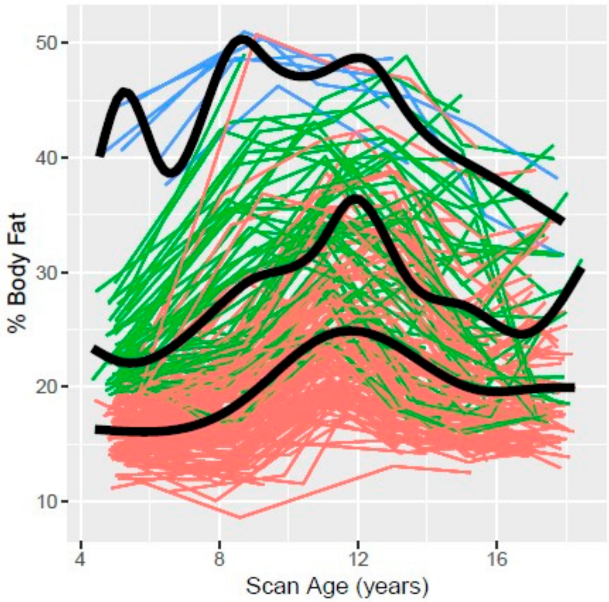

Figure 2b – Females

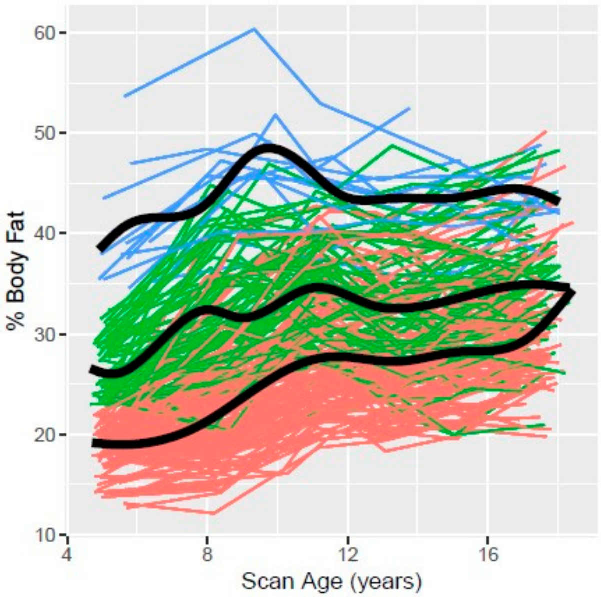

Supplemental Figure 3. Low, middle, and high fat mass index clusters using age 5 year data for Iowa Bone Development Study subjects (n=469, 218 males and 251 females). Orange, green, and blue lines represent individuals assigned to low, middle, and high clusters, respectively.

Figure 3a – Males

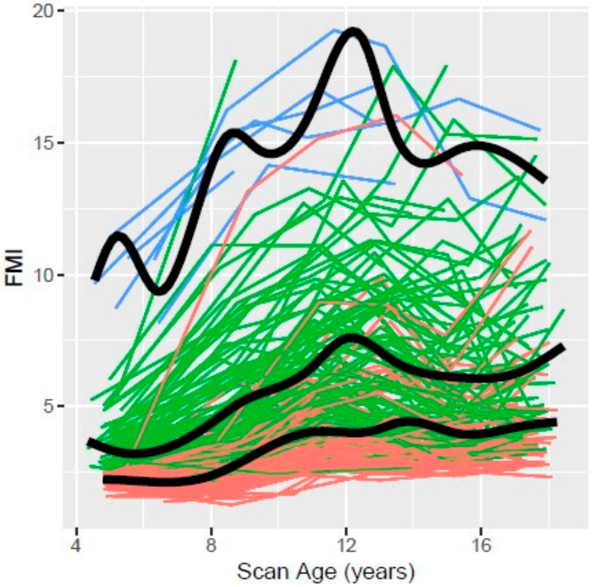

Figure 3b – Females

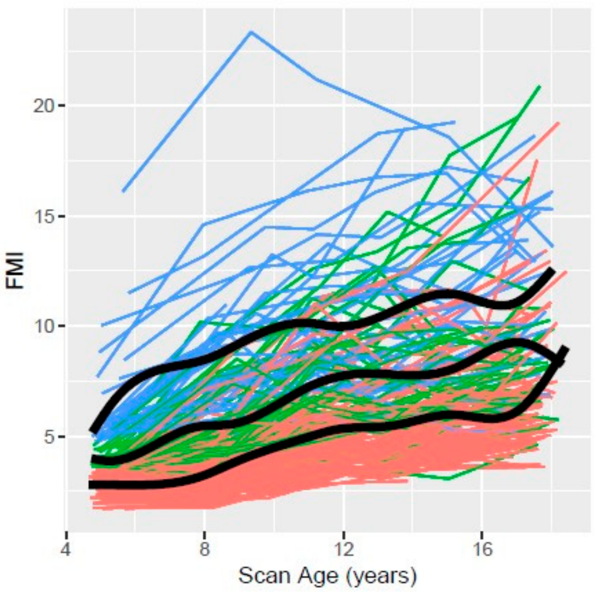

Supplemental Figure 4. Low, middle, and high fat free mass index clusters using age 5 year data for Iowa Bone Development Study subjects (n=469, 218 males and 251 females). Orange, green, and blue lines represent individuals assigned to low, middle, and high clusters, respectively.

Figure 4a – Males

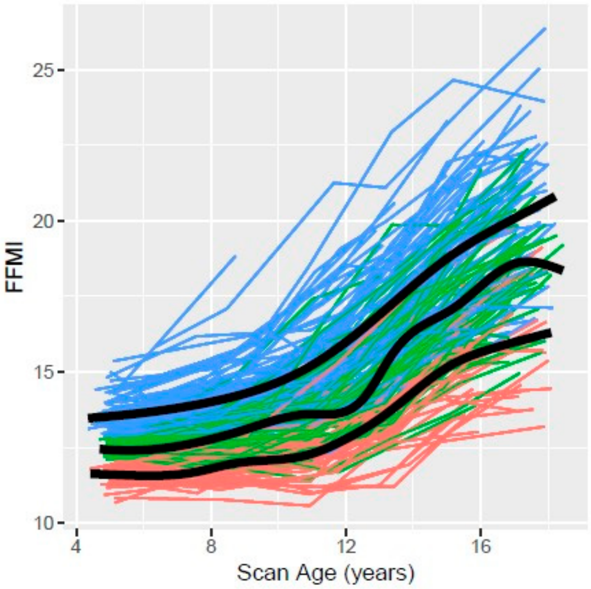

Figure 4b – Females

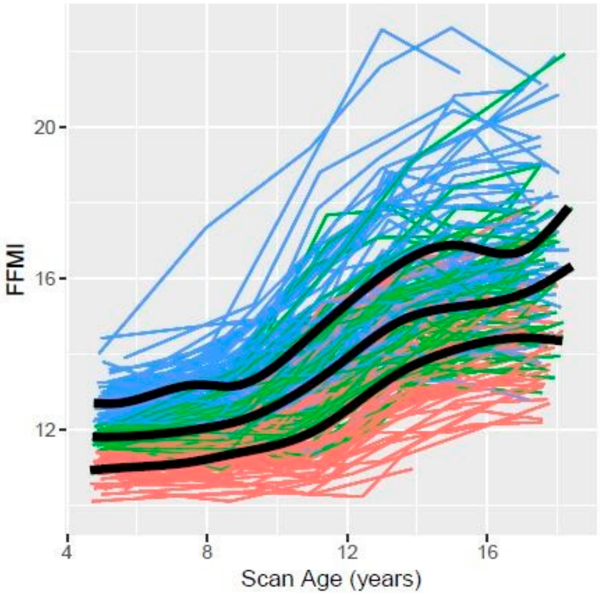

Supplement: Supplementary file 1 [file children-07-00192-s001.pdf]
